# Risky Play and Social Behaviors among Japanese Preschoolers: Direct Observation Method

**DOI:** 10.3390/ijerph19137889

**Published:** 2022-06-27

**Authors:** Natsuko Imai, Akiko Shikano, Tetsuhiro Kidokoro, Shingo Noi

**Affiliations:** 1 Graduate School of Health and Sport Science, Nippon Sport Science University, Tokyo 158-8508, Japan; 2 Research Institute for Health and Sport Science, Nippon Sport Science University, Tokyo 158-8508, Japan; shikano.a@nittai.ac.jp (A.S.); kidokoro@nittai.ac.jp (T.K.); nois@nittai.ac.jp (S.N.)

**Keywords:** risky play, physical activity, SDQ, direct observation, parental employment, Japanese preschoolers

## Abstract

While limited evidence is available, preliminary studies highlight the potential health benefits of risky play. However, most of the studies have used subjective methods (i.e., questionnaires) to evaluate children’s risky play, which limits their validity and reliability. The purpose of the present study was to examine the relationship between the frequency of risky play and social behavior among Japanese preschoolers by using a valid and reliable method such as direct observation. A total of 32 Japanese preschoolers (71.4 ± 3.5 months old) participated in the study, and their social behaviors were measured by the Strength and Difficulties Questionnaire (SDQ). Data regarding the frequency of risky play was collected through direct observation. Results stated that, in a non-adjusted model, there was no significant association between children’s risky play and prosocial behavior. However, the association became significant after adjusting for covariates such as gender, parental employment status, and physical activity. In contrast, there was no significant association between children’s risky play and problem behavior (hyperactivity and aggression) after adjusting for covariates. In conclusion, covariates such as parental employment should be considered when examining the benefits of risky play.

## 1. Introduction

Research has shown that outdoor play in early childhood has a positive impact on the health of children [1,2,3,4]. Frequency of outdoor play is positively associated with lower risk of obesity [5], depression [6], and social skills [7,8]. A follow-up survey conducted in Japan in 2019 by the Ministry of Education, Culture, Sports, Science and Technology (MEXT) showed that young children who graduated from preschools that offered physical fitness-related initiatives, such as exercise play, had a higher frequency of exercise as well as better physical fitness test scores, compared to children who graduated from preschools that did not have them. These results suggest that children who have had a lot of physical activity and play in their early childhood are more likely to be physically active. On the other hand, children reported spending more time at home and more time on screen-based activities in 2015 than 1975 [9]. Similarly in Japan, it has been reported that outdoor play has decreased in 2015 compared to 1995 [10], and there is rising concern about the physical and mental effects of the decrease in outdoor play.

Amidst concerns about the decline in outdoor play, attention has shifted to adventurous play, or “risky play”, defined as “thrilling and exciting forms of physical play that involve uncertainty and the risk of physical disability” [11]. Risky play has been categorized into (1) play with great heights—danger of injury from falling, such as all forms of climbing, jumping, hanging/dangling, or balancing from heights; (2) play with high speed—uncontrolled speed and pace that can lead to a collision with something (or someone), for instance bicycling at high speeds, sledging (winter), sliding, or running (uncontrollably); (3) play with dangerous tools—that can lead to injuries, for instance, axes, saws, knives, hammers, or ropes; (4) play near dangerous elements—where one can fall into or from something, such as water or a fire pit; (5) rough-and-tumble play—where children can harm each other, for instance, wrestling, fighting, or fencing with sticks; (6) play where children go exploring alone—for instance without supervision and where there are no fences, such as in the woods; (7) play with impact—children crashing into something repeatedly just for fun; and (8) vicarious play—children experiencing thrill by watching other children (most often older) engaging in risk [12]. 

Risky play is considered to be an especially active form of play from infancy to early childhood [13]. It has been reported that children who prefer to play with their peers have higher ratings from their peers [14,15,16] and that risky play in preschools and schools is effective in improving children’s risk perception and social skills [17]. Furthermore, some studies have reported that risky experiences in early childhood affect relationships and social skills in adolescence [18]. Thus, it is thought that children who prefer risky play in early childhood develop the ability to overcome difficulties and social skills through such play. While these studies provide important implications, they have only employed subjective methods (i.e., parental report using questionnaire) to evaluate children’s risky play, which limits their validity and reliability [19]. Particularly, children in these age groups are known to change their behaviors in a very short span of time [20]; therefore, questionnaires may not capture their behavior accurately [21]. Direct observation, in contrast, is a method that has proven to be highly valid and reliable, as it can also reveal the transformation of play [22]. 

We examined the frequency of risky plays (i.e., jumping from high places, playing at high speed), which had been examined separately, by combining them and using direct observation methods. A similar study conducted by Sandseter et al. (2021) [19] did not include sociability or difficulty. Thus, the purpose of this study was to examine the relationship between risky play and prosocial behavior, as well as difficulties in daily life, among children.

## 2. Materials and Methods

### 2.1. Ethical Considerations

Written informed consent was provided by the parents of the subjects for their children to participate in the study. The study was conducted in accordance with the Declaration of Helsinki and approved by the Institutional Ethics Advisory Committee of the Nippon Sport Science University (Approval No. 019-H081).

### 2.2. Participants

This study was conducted in October 2021, with 38 children (71.4 ± 3.5 month) and their parents attending one public kindergarten in Tokyo, Japan. Of the 38 subjects, 32 children (valid data = 84.2%) who were observed for all three days of the free play observation period were included in the analysis. Six children who were absent from kindergarten during the study period were excluded from the analysis. The daily schedule of the children in the preschool is shown in Table 1. The kindergarten visited in this study was a typical preschool that was set up with free space in addition to fixed playground equipment such as swings, jungle gym, iron bars, climbing bars, and a sandbox.

### 2.3. Study Design 

This is a cross-sectional study which examines the association between risky play and prosocial behaviors among preschool children. Social behaviors were measured by the Strength and Difficulties Questionnaire (SDQ) [23], while risky play was measured by the Risky Play Scale [24]. Covariates were measured through direct observation. 

In this study, risky play is as an independent variable, while social behavior is the dependent variable. Physical activity intensity and parental employment status were determined as covariates. Details are as follows.

**Figure 1:**
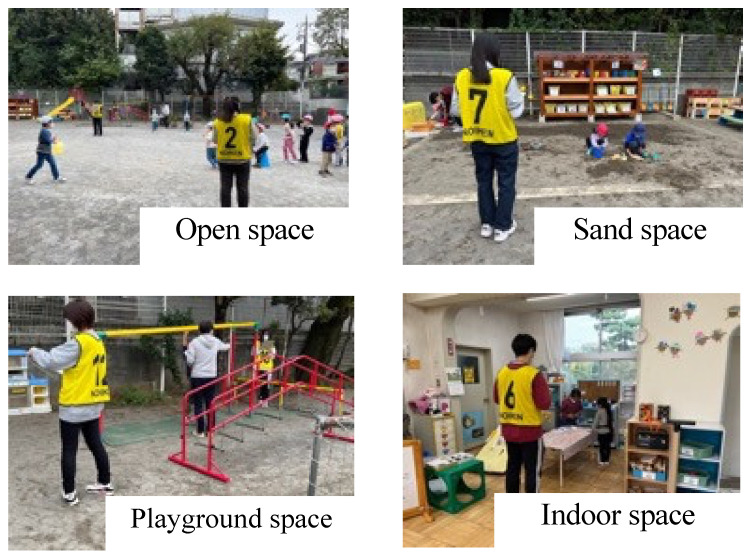
Observation of play in each area.

1. Social behaviors
  The 25-item parental rating form of the SDQ (Japanese version) was used as the objective variable for prosocial behavior and difficulties in daily life [25]. SDQ had been shown to be a valid and reliable questionnaire [23], and the Japanese version of SDQ was also confirmed to be valid and reliable among Japanese children [25]. The SDQ consists of five subscales: “prosocial behavior”, “hyperactivity/inattention”, “problem behavior”, “emotional instability”, and “friendship problems”. Parents were asked to answer each item using a three-point scale from “not applicable (0 point)” to “applicable (2 points)”. In this study, the “total difficulty score”, which is the sum of the “prosocial behavior score” and the four subscales excluding it, was used. The higher the prosocial behavior score, the more prosocial the behavior. In contrast, the range of the overall difficulty score is 0–40, with higher scores indicating more difficult behaviors. Previous studies have confirmed good internal consistency of the overall difficulty scores (Cronbach’s alpha for the scale was 0.78 [26]).
2. Risky play
  The data were collected by four trained observers following a strict protocol using the nature observation method [27]. Before the data collection, the observers had standardized sessions to make sure their evaluation was consistent among the observers. More specifically, the observers were asked to evaluate the randomly collected children’s play. When the observers’ evaluations differed, the observers discussed and reached a consensus to ensure consistency among the observers.
  The risky play observations were conducted during free play time (12:30–13:30) from 25–27 October 2021 (3 days). In this study, a time sampling method [28] of 30 s/time per children was used. Measurements were taken by observing the activities during free play in each of the four play areas (open space, playground space, sand space, and indoor space) (Figure 1). At first, the observers were asked to record children’s free play every 30 s. Thereafter, children’s play were categorized “risky play” or “non-risky play” using the Risky Play Scale [24] which was validated by previous studies [13,29,30,31]. In the Risky Play Scale, there were six subcategories (i.e., great heights, high speed, dangerous tools, dangerous elements, disappear/get lost, and rough and tumble play) and children’s behavior were also categorized using the subscale.
3. Covariates
  Previous studies have reported that physical activity and parental factors influence young children’s risky play and social skills [32,33,34,35]. In this study, we collected data on physical activity and the parental employment status. Physical activity was measured by a triaxial accelerometer (ActiGraph wGT3X-BT, LLC, Pensacola, FL, USA). Accelerometers are proven to be valid and reliable activity monitors for measuring physical activity in children [36,37]. Participants were asked to wear the accelerometer on the right side of their waist using a belt, for seven consecutive days (Monday through Sunday), except during sleep or water-based activities (e.g., showering or swimming). Data were collected in 15-s epochs. Non-wear time was defined as a period of more than 60 min of continuous zero counts recorded in ActiGraph [38]. Only participants with at least 10 h of wear per day and a minimum of 4 days (including at least one weekend) were included in the analysis [39]. For each participant, the mean MVPA (min/day) was calculated. The cutoff points from Evenson et al. (2008) [36] were selected to determine the time spent on MVPA. MVPA time was calculated as mean daily minutes ≥ 2296 counts/min from all valid days. Data collected were stored in ActiLife software version 6.13.3 (ActiGraph, LLC, Pensacola, FL, USA). Parental employment status was asked of the parents by means of a questionnaire. These were defined as “Single-income family” when one of the parents worked, and “Dual-income family” when both parents or one parent in a single-parent household worked. Full-time and part-time workers were not considered.

### 2.4. Statistical Analyses

To examine the differences in the prevalence of risk play between boys and girls, independent *t*-tests were performed. The relationship between risky play and social behaviors was examined by using logistic regression, with risk play being an explanatory variable, and prosocial behaviors and total difficulty score being an outcome variable. Previous studies have reported that the frequency of implementation of risky play is 10–20% of the total play, which is low [24,31]. For this reason, we decided to use the cut-point based on the prevalence of risky play in our sample. Here, those who performed risky play at least once during the observation period were assigned “1”, those who did not perform risky play were assigned “0”. MVPA longer than the median was assigned “1”, and those whose MVPA was shorter than the median was assigned “0”. An analysis of covariance was conducted using risky play (group with risky play = 1, group without risky play = 0) as the explanatory variable, and the prosocial behavior score and total difficulty score as the objective variables (Model 1). We also conducted an analysis of covariance adjusting for the effects of gender and parental employment status (Model 2), and an analysis of covariance adjusting for the effects of MVPA in addition to gender and parental employment status (Model 3).

IBM SPSS^®^ ver. 26 (IBM Corp., Armonk, NY, USA) was used for these series of statistical processing, and statistical significance was determined at a level of less than 5% risk.

## 3. Results

### 3.1. Prevalence of Risky Play

In total, 271 free play incidents were extracted from the activities of the children and the number of observations per participant child over a 3-day period was 8.1 ± 2.3 times (Table 2). The types of play and the incidence of risky play were also examined. The results showed that tag was the most frequent activity for boys and playground equipment was the most frequent activity for girls.

The incidence of risky play was 12% of all observations. The most frequently observed risky play was “High speed”, while “Dangerous tools”, “Dangerous elements”, and “Disappear/get lost” were never observed. Furthermore, 11 of the 32 children in the study never performed risky play.

### 3.2. Relationship between Risky Play and Social Behaviors

**Figure 2:**
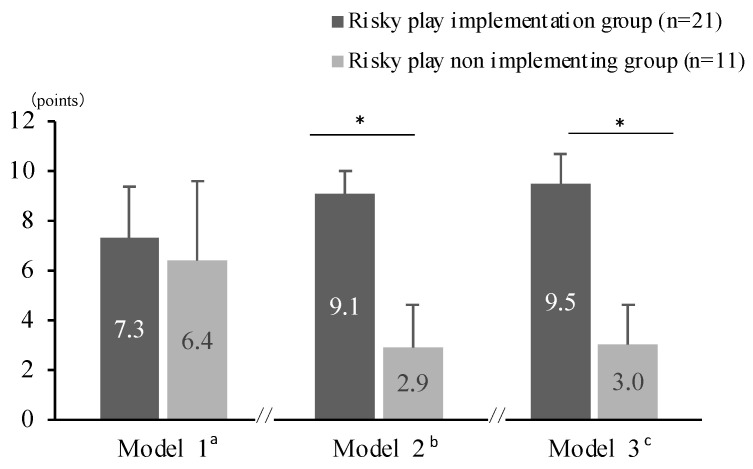
Prosocial scores by implementation of risky play. (**a**) Results of covariance analysis with explanatory variable as presence of risky play and objective variable as prosocial score (F(1, 29) = 0.84, *p* = 0.37, η^2^ = 0.03). (**b**) Results of covariance analysis with explanatory variable as presence or absence of risky play, objective variable as prosocial score, and adjustment variables as gender and type of employment (F(1, 27) = 6.11, *p* = 0.02, η^2^ = 0.23). (**c**) Results of covariance analysis with explanatory variable as presence or absence of risky play, objective variable as prosocial score, and adjustment variables as gender, type of employment and MVPA. (F(1, 22)= 5.76, *p* = 0.03, η^2^ = 0.26) * *p* < 0.05.

In a non-adjusted model, there was no significant association between children’s risky play and prosocial behavior (Model 1). However, the association became significant after adjusting for covariates including gender and parental employment status (Model 2). Additionally, the associations remained significant after additionally adjusting for MVPA (Model 3) (Figure 2). The results showed that the model with no covariate input (Model 1), showed no association between risky play and prosocial behavior. However, Model 2, which adjusted for gender and parental employment status, and Model 3, which adjusted for MVPA, showed a positive association between the two models.

**Figure 3:**
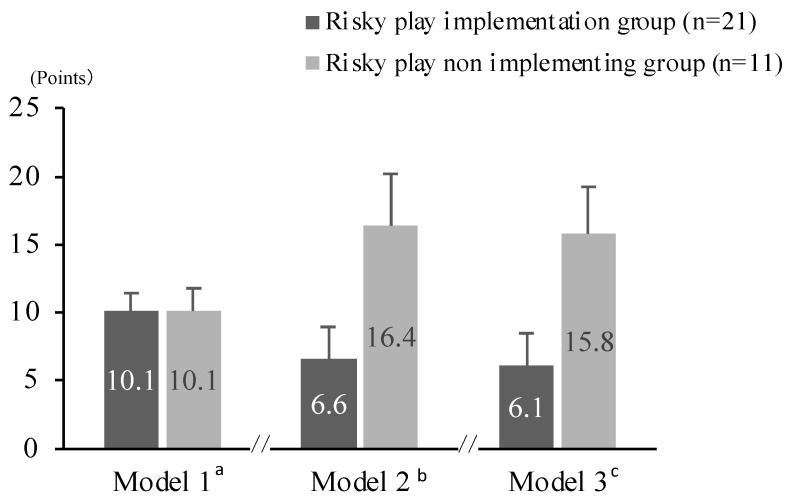
Total difficulty scores by implementation of risky play. (**a**) Results of covariance analysis with explanatory variable as presence of risky play and objective variable as total difficulty score (F(1, 29) = 0.00, *p* = 0.99, η^2^ = 0.00). (**b**) Results of covariance analysis with risky play as explanatory variable, total difficulty as objective variable, and gender and employment status as adjustment variables (F(1, 27) = 2.92, *p* = 0.10, η^2^ = 0.11). (**c**) Results of covariance analysis with explanatory variable as presence or absence of risky play, objective variable as total difficulty score, and adjustment variables as gender, type of employment and MVPA (F(1, 22)= 2.46, *p* = 0.13, η^2^ = 0.11).

Figure 3 shows the results of the analysis of covariance with risky play as the explanatory variable and the total difficulty score as the objective variable. In the non-adjusted model, there was no significant association between children’s risky play and problem behaviors (Model 1). In this case, no significant associations were found after adjusting for covariates such as gender and parental employment status, as well as additional adjustment for MVPA (Model 3).

## 4. Discussion

In this study, we examined the relationship between risky play and social behaviors by directly observing free play situations in early childhood. The results showed a significant positive association between risky play and prosocial behavior after adjusting for gender and parental employment status. Furthermore, a similar positive association between risky play and prosocial behavior was found after adjusting for MVPA. Thus, children who engage in risky play in early childhood tend to engage in prosocial behaviors as well. However, it was found that problem behaviors were not associated with risky play. This is the first study to show that the frequency of risky play is positively associated with prosocial behavior among preschool children by employing direct observation. 

### 4.1. Facts about Risky Play

In this study, we directly observed risky play on free play surfaces to understand the reality. As a result, the incidence of risky play observed in this study was 12% of the total which was lower than previously reported [24]. Sandseter et al. (2021) [31] who observed risky play during free play time in early childhood and examined its incidence, reported that 20% of all play was risky play. We speculate that these differences in results were influenced by differences in the location of observation. Previous studies have reported that parents and caregivers tend to regulate their children’s outdoor activities due to concerns about safety and security issues [17]. Therefore, it is possible that the children in this study were in a situation where the presence of a caregiver made it difficult to engage in risky play, even though they were in a free play environment. 

Furthermore, cultural differences have also been noted to influence risky play [31]. Differences may have been observed depending on the level of tolerance for risky play in each country. Indeed, parents and caregivers in Norway and Canada are less likely than parents and caregivers in the United States and Australia to restrict children’s risky play and to allow them to play outdoors by themselves [40,41]. Although none of the research findings on risky play in Japan are available and cannot be directly compared, considering the recent decline in outdoor play in Japan due to concerns about injuries and security issues [7], Japanese parents and caregivers tend to be risk-averse too. This attitude on the part of parents and caregivers may have contributed to the low rate of risky play among children.

### 4.2. Relationship between Risky Play and Social Behaviors

This study indicated that children who engage in risky play in preschool developed prosocial behavior, such as helping their peers and helping clean up. These results are consistent with previous research suggesting that risky play is beneficial for social skills [42,43,44]. This association between risky play and prosocial behavior is influenced by several potential mechanisms. For example, it has been reported that rough-and-tumble play enhances the social skills of boys and the ones who prefer rough-and-tumble play are more popular with their peers [45]. It has also been suggested that while engaging in risky play, the behavior of helping peers to overcome barriers improves interpersonal and social skills [17]. Therefore, the results of this study suggest that exposure to danger, conflict, and challenge through risky play may lead to the development of social skills through the development of behaviors that prioritize helping others. In addition, there was an association between gender and parental employment status in Model 2, which was not observed in Model 1 without confounding factors. These results are consistent with previous findings that boys play riskily more frequently than girls [46,47] and that the social environmental factor—especially parents’ work status—affects infants’ play experiences [11,48,49]. Although previous studies have examined the relationship between risky play and social factors such as children’s residential environment, convenience, and parental supervision [50,51], few studies have examined the relationship between risky play and prosocial behavior, taking these factors into account. Therefore, this study is quite significant, as it takes confounding factors into consideration. By contrast, as Figure 2 shows, there is no significant difference between the prosocial behavior scores of Model 3, which adjusted for MVPA in addition to gender and parental employment status, and Model 2, which adjusted only for gender and work status. Poitras et al. (2016) [52] found that the association between physical activity intensity and prosocial behavior and social behavior was limited, and that the association was weak. This suggests that regardless of the amount of MVPA during the day, the content of the risky play may have affected prosocial behavior.

Alternatively, results examining the relationship between risky play and problem behavior did not confirm any significant association in either model (Figure 3). Although several studies have examined the association between rough-and-tumble play and aggression and hyperactive behavior, they have shown inconsistent results. For example, Pellegrini (1989) [45], who examined the association between rough-and-tumble play and the frequency of aggression, reported no association between them for either gender. By contrast, some studies have indicated that rough-and-tumble play at home can lead to aggression [14]. This disparity in reviews may be due to differences in the sample size. In fact, as Figure 3 shows, although there was no significant association between the risky play group and the non-play group in Models 2 and 3, the effect size of the difference was large. Therefore, it cannot be denied that an association between the two groups could have been observed in this study if the number of subjects had been increased. Future studies with a larger number of participants are needed.

### 4.3. Strengths and Limitations

One of the main strengths of this study is that it directly observed risky play by category. Young children’s play is constantly changing and the fact that we were able to directly observe each of these scenes was important in capturing the reality of children’s risky play. We also considered gender and parental employment status as relevant factors. As mentioned, outdoor play in early childhood has decreased, suggesting that socio-environmental factors are influencing this trend. Considering the current situation, it was crucial that we examined the relationship between risky play and social skills, taking different factors into account.

However, the following limitations of this study can be addressed in future research. The problem of selection bias was a limitation because we could only recruit a small sample of children from one school, which limits the generalizability of our results. In addition, a possible reason for the lack of association between risky play and problem behavior—including hyperactivity and aggression—in the present study, could be the effect of a time lag between the onset of spontaneous behavior and the effect of risky play on behavioral inhibition. In fact, previous studies have shown that there is a main effect of age on behavioral inhibition in young children and that it develops with increasing age [53]. Therefore, future studies with more subjects and measurements at different ages are required. Lastly, this is a cross-sectional study which does not examine the direction of causality of the hypothesized model and its temporal effects. Therefore, it is necessary to conduct a longitudinal study to clarify the temporal relationship.

## 5. Conclusions

This is the first study to examine the association between risky play and prosocial behavior among preschool children using direct observation. In a non-adjusted model, there was no significant association between children’s risky play with prosocial behavior. However, it became significant after adjusting for covariates including gender, parental employment status, and physical activity. In contrast, there was no significant association between children’s risky play and hyperactive behavior after adjusting for covariates. The present study revealed that significant associations of risky play with prosocial behavior were only confirmed after adjusting for covariates, including parental employment. 

## Figures and Tables

**Table 1:** Kindergarten daily schedule.

Time	Schedule
~9:00	Going to kindergarten
~10:45	Free play time
~11:40	All together childcare
12:00~12:30	Lunch
~13:30	Free play time
14:00~	Going home from kindergarten

**Table 2:** Descriptive characteristics of the participants.

	All (n = 32)	Boys (n = 21)	Girl (n = 11)	Comparisons, *p*-Value (Boys vs. Girls)
Basic characteristics				
Age (month)	71.4 ± 3.5	71.3 ± 3.7	71.6 ± 3.2	*p* = 0.208
Risky play				
Risky play implementation rate (%)	12.1	14.2	9.8	*p* < 0.001 *
Great heights (%)	2.1	2.8	1.6	
High speed (%)	8.6	9.6	7.1	
Dangerous tools (%)	0	0	0	
Dangerous elements (%)	0	0	0	
Disappear/get lost (%)	0	0	0	
Rough and Tumble play (%)	1.4	1.8	1.1	
Strength and Difficulties Questionnaire				
Prosociality scores (points)	6.9 ± 2.6	7.0 ± 2.5	6.9 ± 2.8	*p* = 0.959
Total difficulty score (points)	10.1 ± 5.7	10.5 ± 5.0	9.1 ± 7.0	*p* = 0.522

* *p* < 0.001

## Data Availability

The data that support the findings of this study are available from the corresponding author, upon reasonable request.

## References

[1] Tillmann S., Tobin D., Avison W., Gilliland J. (2018). Mental health benefits of interactions with nature in children and teenagers: A systematic review. J. Epidemiol. Community Health.

[2] McCurdy L.E., Winterbottom K.E., Mehta S.S., Roberts J.R. (2010). Using nature and outdoor activity to improve children’s health. Curr. Probl. Pediatr. Adolesc. Health Care.

[3] McCormick R. (2017). Does access to green space impact the mental well-being of children: A systematic review. J. Pediatr. Health Care.

[4] Johnstone A., Hughes A.R., Martin A., Reilly J.J. (2018). Utilising active play interventions to promote physical activity and improve fundamental movement skills in children: A systematic review and meta-analysis. BMC Public Health.

[5] Poulain T., Sobek C., Ludwig J., Igel U., Grande G., Ott V., Kiess W., Körner A., Vogel M. (2020). Associations of Green Spaces and Streets in the Living Environment with Outdoor Activity, Media Use, Overweight/Obesity and Emotional Wellbeing in Children and Adolescents. Int. J. Environ. Res. Public Health.

[6] Rodriguez-Ayllon M., Cadenas-Sánchez C., Estévez-López F., Muñoz N.E., Mora-Gonzalez J., Migueles J.H., Molina-García P., Henriksson H., Mena-Molina A., Martínez-Vizcaíno V. (2019). Role of physical activity and sedentary behavior in the mental health of preschoolers, children and adolescents: A systematic review and meta-analysis. Open Access J. Sports Med..

[7] Loukatari P., Matsouka O., Papadimitriou K., Nani S., Grammatikopoulos V. (2019). The Effect of a Structured Playfulness Program on Social Skills in Kindergarten Children. Int. J. Instr..

[8] Carson V., Lee E.-Y., Hesketh K.D., Hunter S., Kuzik N., Predy M., Rhodes R.E., Rinaldi C.M., Spence J.C., Hinkley T. (2019). Physical activity and sedentary behavior across three time-points and associations with social skills in early childhood. BMC Public Health.

[9] Mullan K. (2019). A child’s day: Trends in time use in the UK from 1975 to 2015. Br. J. Sociol..

[10] Benesse Educational Research and Development Institute, Fifth Infant Life Survey Report. https://berd.benesse.jp/up_images/research/YOJI_all_P01_65.pdf.

[11] Sandseter E.B.H., Kennair L.E.O. (2011). Children’s risky play from an evolutionary perspective: The anti-phobic effects of thrilling experiences. Evol. Psychol..

[12] Sandseter E.B.H., Kleppe R., Tremblay R.E., Boivin M., Peters R.D., Brussoni M. (2019). Outdoor Risky Play. Encyclopedia on Early Childhood Development.

[13] Kleppe R., Melhuish E., Sandseter E.B.H. (2017). Identifying and characterizing risky play in the age one-to-three years. Eur. Early Child Educ. Res. J..

[14] Pellegrini A.D. (1988). Elementary-school children’s rough-and-tumble play and social competence. Dev. Psychol..

[15] DeWolf D.M. (1999). Preschool Children’s Negotiation of Intersubjectivity during Rough-and-Tumble Play.

[16] Colwell M.J., Lindsey E.W. (2005). Preschool children’s pretend and physical play and sex of play partner: Connections to peer competence. Sex Roles.

[17] Dodd H.F., Lester K.J. (2021). Adventurous play as a mechanism for reducing risk for childhood anxiety: A conceptual model. Clin. Child Fam. Psychol. Rev..

[18] Gardner M., Steinberg L. (2005). Peer influence on risk taking, risk preference, and risky decision making in adolescence and adulthood: An experimental study. Dev. Psychol..

[19] Sirard J.R., Pate R.R. (2001). Physical activity assessment in children and adolescents. Sports Med..

[20] Parten M.B. (1932). Social participation among pre-school children. J. Abnorm. Soc. Psychol..

[21] Arney F.M. (2004). A Comparison of Direct Observation and Self-Report Measures of Parenting Behaviour. Ph.D. Thesis.

[22] Pellegrini A. (2001). The role of direct observation in the assessment of young children. J. Child Psychol. Psychiatry.

[23] Goodman A., Lamping D.L., Ploubidis G.B. (2010). When to use broader internalising and externalising subscales instead of the hypothesised five subscales on the Strengths and Difficulties Questionnaire (SDQ): Data from British parents, teachers and children. J. Abnorm. Child Psychol..

[24] Sandseter E.B.H., Sando O.J., Kleppe R. (2021). Associations between children’s risky play and ECEC outdoor play spaces and materials. Int. J. Environ. Res. Public Health.

[25] Iida Y., Moriwaki A., Komatsu S., Kamio Y., Kamio Y. (2014). Standardization of Strength and Difficulties Questionnaire based on parents’ and teachers’ ratings among preschool children (4-5 years old) in Japan. Prevalence and Developmental Changes of Developmental Disorders in Pre- and Post-School Children: A Community-Based Cross-Sectional and Longitudinal Study.

[26] Theunissen M.H., Vogels A.G., de Wolff M.S., Reijneveld S.A. (2013). Characteristics of the strengths and difficulties questionnaire in preschool children. Pediatrics.

[27] Smith P.K. (2011). Observational Methods in Studying Play.

[28] Altmann J. (1974). Observational study of behavior: Sampling methods. Behaviour.

[29] Hansen Sandseter E.B. (2007). Categorising risky play—How can we identify risk-taking in children’s play?. Eur. Early Child. Educ. Res. J..

[30] Sandseter E.B.H. (2009). Characteristics of risky play. J. Adventure Educ. Outdoor Learn..

[31] Hansen Sandseter E.B., Kleppe R., Sando O.J. (2020). The Prevalence of Risky Play in Young Children’s Indoor and Outdoor Free Play. Early Child. Educ. J..

[32] Aarts M.J., Wendel-Vos W., van Oers H.A., Van de Goor I.A., Schuit A.J. (2010). Environmental determinants of outdoor play in children: A large-scale cross-sectional study. Am. J. Prev. Med..

[33] Boxberger K., Reimers A.K. (2019). Parental correlates of outdoor play in boys and girls aged 0 to 12—A systematic review. Int. J. Environ. Res. Public Health.

[34] Guryan J., Hurst E., Kearney M. (2008). Parental education and parental time with children. J. Econ. Perspect..

[35] Noi S., Shikano A., Tanaka R., Tanabe K., Enomoto N., Kidokoro T., Yamada N., Yoshinaga M. (2021). The pathways linking to sleep habits among children and adolescents: A complete survey at Setagaya-ku, Tokyo. Int. J. Environ. Res. Public Health.

[36] Evenson K.R., Catellier D.J., Gill K., Ondrak K.S., McMurray R.G. (2008). Calibration of two objective measures of physical activity for children. J. Sports Sci..

[37] Freedson P., Pober D., Janz K.F. (2005). Calibration of accelerometer output for children. Med. Sci. Sports Exerc..

[38] Masse L.C., Fuemmeler B.F., Anderson C.B., Matthews C.E., Trost S.G., Catellier D.J., Treuth M. (2005). Accelerometer data reduction: A comparison of four reduction algorithms on select outcome variables. Med. Sci. Sports Exerc..

[39] Trost S.G., Pate R.R., Freedson P.S., Sallis J.F., Taylor W.C. (2000). Using objective physical activity measures with youth: How many days of monitoring are needed?. Med. Sci. Sports Exerc..

[40] Watchman T., Spencer-Cavaliere N. (2017). Times have changed: Parent perspectives on children’s free play and sport. Psychol. Sport Exerc..

[41] Little H., Sandseter E.B.H., Wyver S. (2012). Early childhood teachers’ beliefs about children’s risky play in Australia and Norway. Contemp. Issues Early Child.

[42] Brussoni M., Gibbons R., Gray C., Ishikawa T., Sandseter E.B., Bienenstock A., Chabot G., Fuselli P., Herrington S., Janssen I. (2015). What is the relationship between risky outdoor play and health in children?. A systematic review. Int. J. Environ. Res. Public Health.

[43] Prezza M., Pilloni S., Morabito C., Sersante C., Alparone F.R., Giuliani M.V. (2001). The influence of psychosocial and environmental factors on children’s independent mobility and relationship to peer frequentation. J. Community Appl. Soc. Psychol..

[44] Gull C., Goldenstein S.L., Rosengarten T. (2018). Benefits and Risks of Tree Climbing on Child Development and Resiliency. Early Child. Educ. J..

[45] Pellegrini A.D. (1989). Elementary school children’s rough-and-tumble play. Early Child. Res. Q..

[46] Little H., Eager D. (2010). Risk, challenge and safety: Implications for play quality and playground design. Eur. Early Child Educ. Res. J..

[47] Morrongiello B.A., Kane A., Zdzieborski D. (2011). “I think he is in his room playing a video game”: Parental supervision of young elementary-school children at home. J. Pediatr. Psychol..

[48] Lee E.Y., Bains A., Hunter S., Ament A., Brazo-Sayavera J., Carson V., Hakimi S., Huang W.Y., Janssen I., Lee M. (2021). Systematic review of the correlates of outdoor play and time among children aged 3-12 years. Int. J. Behav. Nutr. Phys. Act..

[49] Brussoni M., Olsen L.L., Pike I., Sleet D.A. (2012). Risky play and children’s safety: Balancing priorities for optimal child development. Int. J. Environ. Res. Public Health.

[50] Jelleyman C., McPhee J., Brussoni M., Bundy A., Duncan S. (2019). A cross-sectional description of parental perceptions and practices related to risky play and independent mobility in children: The New Zealand state of play survey. Int. J. Environ. Res. Public Health.

[51] McFarland L., Laird S.G. (2018). Parents’ and early childhood educators’ attitudes and practices in relation to children’s outdoor risky play. Early Child Educ. J..

[52] Poitras V.J., Gray C.E., Borghese M.M., Carson V., Chaput J.P., Janssen I., Katzmarzyk P.T., Pate R.R., Connor Gorber S., Kho M.E. (2016). Systematic review of the relationships between objectively measured physical activity and health indicators in school-aged children and youth. Appl. Physiol. Nutr. Metab..

[53] Brocki K.C., Bohlin G. (2004). Executive functions in children aged 6 to 13: A dimensional and developmental study. Dev. Neuropsychol..

